# Metal transfer and bead formation in plasma arc–based wire arc additive manufacturing with vertical wire feeding

**DOI:** 10.1007/s00170-025-15793-4

**Published:** 2025-06-04

**Authors:** Chong Wang, Xin Chen, Wojciech Suder, Jialuo Ding, Goncalo Pardal, Stewart Williams

**Affiliations:** Welding and Additive Manufacturing Centre, Cranfield University, Bedfordshire, MK43 0 AL UK

**Keywords:** Directed energy deposition, Deposition rate, Bead formation, Keyhole behaviour, Metal transfer

## Abstract

Wire arc additive manufacturing (WAAM) is suitable for building large-scale engineering structures with high deposition rates and relatively low costs. However, in a typical plasma transferred arc (PTA)–based WAAM process using an inclined wire and vertical torch, keyhole defects can occur due to the high arc pressure, and the process is sensitive to the wire-feeding position with respect to the workpiece. Therefore, in this study, a PTA-based WAAM process with a new configuration employing a vertical wire and an inclined plasma torch was investigated for the potential of mitigation of keyhole formation and improvement of process tolerance. In particular, detailed investigations were carried out on the metal transfer mechanisms and bead formation characteristics under various processing conditions. The results show that the new configuration significantly reduces the likelihood of keyhole formation compared with the conventional approach due to the changes in arc pressure and heat distribution. Systematic analysis reveals that process parameters, including wire feed speed, arc current, and plasma gas flow rate, strongly influence droplet transfer stability, melt pool dynamics, and final bead morphology, which offer guidance for future process optimisation.

## Introduction

Wire arc additive manufacturing (WAAM) has experienced rapid development over the last two decades, emerging as a versatile process for the fabrication of medium to large metallic components [1–3]. Compared to traditional subtractive manufacturing where components are machined from forged billets, WAAM significantly reduces material wastage and lead time [4]. Within the WAAM process, an electric arc, including gas metal arc (GMA) [5], gas tungsten arc (GTA) [6], and plasma transferred arc (PTA) [7], is employed as a heat source to melt the metallic wire. PTA is a preferred option for printing high value-added materials, such as titanium- and nickel-based alloys [8–10], due to the higher process stability compared to GMA and higher process tolerance compared to GTA.

In conventional PTA-based WAAM, the plasma torch is placed vertically, and the wire is fed at an inclined angle; the deposition process is sensitive to the positioning of the wire relative to the workpiece [11, 12]. For example, positioning the wire too high above the workpiece results in the generation of excessive spatters. Conversely, placing the wire too low could lead to the wire piercing the cold material at the front of the melt pool, inducing instability or, in extreme cases, misalignment of the wire. Therefore, there is a need for a stable PTA deposition process that remains unaffected by variations in the wire positioning.

In addition, keyhole formation has been recognised as a major challenge that compromises bead quality and limits the productivity in conventional PTA-based WAAM [11]. Previous studies have explored various strategies to mitigate keyhole defects, such as plasma gas composition [13], optimisation of process parameters [11], and alternative wire-feeding methods [14]. These approaches primarily aim to stabilise the molten pool dynamics by redistributing arc pressure or modifying heat input. In particular, numerical modelling efforts [15, 16] have contributed to a better understanding of arc pressure distributions and their influence on keyhole behaviour. However, the interaction effects between wire-feeding direction and arc incidence angle have received limited attention.

Furthermore, in contrast to the omnidirectional nature of the GMA-based process, where consistent bead shapes can be attained through various travel directions, the conventional PTA deposition process results in distinct bead shapes depending on the torch leading or trailing position employed. Therefore, a PTA process with a vertical wire and inclined torch is proposed to increase the process tolerance and deposit quality. With vertical wire feeding, the process should not be affected by the relative position between the wire and workpiece. It is also expected that the arc column with an inclined torch will be spread over a larger area compared to a vertical torch, which will reduce the arc pressure and thereby reduce the likelihood of keyhole formation. Furthermore, the omnidirectional characteristics of the process are unclear. Therefore, it is crucial to acquire a comprehensive understanding of the PTA-based WAAM using a vertical wire.

Research has been carried out to achieve consistent bead formations across various wire positions in wire-based additive manufacturing (AM). Wu et al. [17] studied the effect of wire-feeding direction and wire-feeding angle on GTA-based WAAM. They found that the bead geometry varied considerably in different wire-feeding directions at wire-feeding angles of 30–50° and 70°, but a uniform bead shape could be obtained at a wire-feeding angle of 60° in any direction. It is noteworthy that the process window in their study was very narrow, and uniform deposits cannot be achieved after changing any process variables. To address the limitations associated with conventional lateral wire feeding, Wang et al. [18] developed a laser wire setup, integrating the laser and wire coaxially. The results showed that a uniform bead shape with a smooth surface was obtained with various travel directions. Fu et al. [19] proposed a laser deposition process utilising a vertical wire and three axisymmetric inclined lasers. The successful deposition of a complex component with a consistent bead shape showcased the advantages of a vertical wire setup. Similarly, Huft [20] developed a PTA deposition process that combines a vertical wire and three axisymmetric inclined torches, significantly reducing the sensitivity of the process to travel direction. However, this process is complex due to the interactions between the arcs. In addition, the high remelting caused by the high energy input from the three torches resulted in low process efficiency.

Compared to a vertically oriented torch, the deployment of an inclined torch introduces an alteration in the arc profile. This modification gives rise to variations in several factors linked to the heat source, including energy distribution and arc pressure. The energy distribution of the arc affects the melting dynamics of wire, thereby exerting a direct influence on process productivity. Enhancing the efficiency of wire melting corresponds to an increase in productivity, which is a desirable outcome in the realm of production. In terms of arc pressure, it plays a dominant role in keyhole formation and metal transfer behaviour. The metal transfer has an important effect on the process stability and bead formation. In the deposition process, the metal transfer is determined by different forces acting on the droplet, and any alterations induce corresponding changes in the resultant force, consequently influencing the behaviour of metal transfer and the appearance of the final bead [21]. Therefore, it is important to investigate the metal transfer behaviour in the new configuration.

In this study, a PTA-based WAAM process with a vertical wire and inclined torch was studied for the deposition of Ti-6 Al-4 V. The effect of torch position on bead shape was studied by experiments, and the mechanism of bead formation was revealed by a computational fluid dynamics (CFD) model. In addition, the keyhole formation and wire melting behaviour were studied and compared with those in the conventional configuration. Furthermore, the metal transfer behaviour in the new configuration and the effect of different process parameters, including wire feed speed (WFS), arc current, and plasma gas flow rate on the metal transfer and bead quality, were investigated.

## Methodology

### Materials and setup

**Fig. 1:**
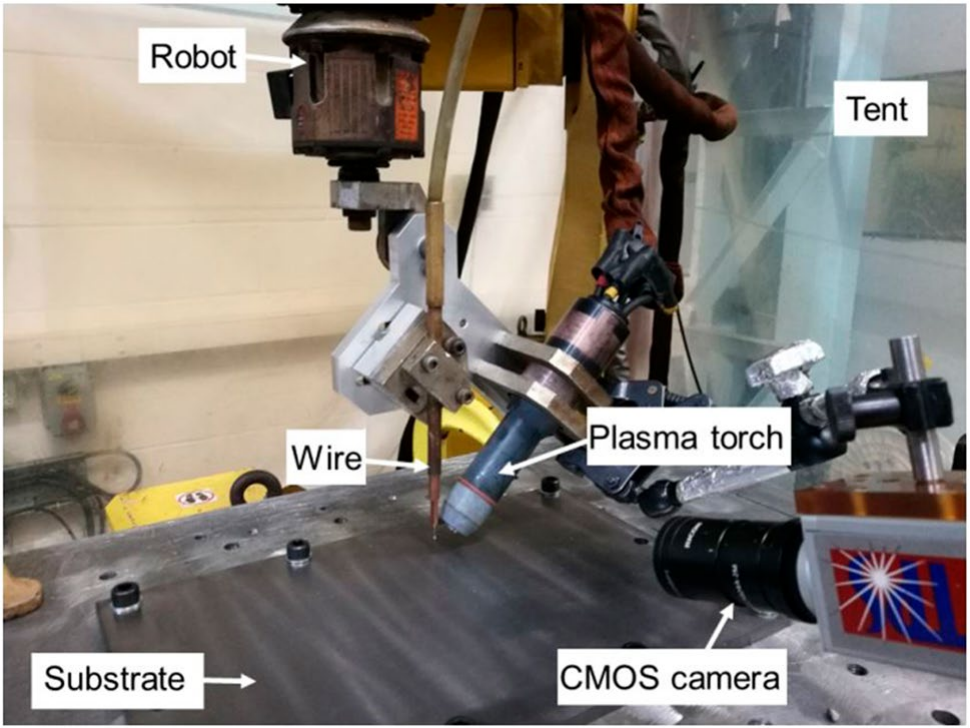
Experimental setup for the PTA-based WAAM system featuring a vertical wire and inclined torch

Figure 1 shows the experimental setup for the new PTA configuration featuring a vertical wire and inclined torch. A 1.6-mm Ti-6 Al-4 V wire was used as feedstock, which was fed perpendicularly to the substrate. The substrate used was also Ti-6 Al-4 V with dimensions of 300 mm × 200 mm × 7 mm, which was securely clamped onto an aluminium baseplate. For the WAAM system, a six-axis Fanuc robot and a plasma power source (EWM Tetrix 352) were used. The plasma torch and wire were attached to the robot using customised brackets, where the angle of the torch and the position of the wire were adjustable. Both plasma gas and shielding gas used for the plasma torch were pure argon. The deposition process was conducted within a flexible tent filled with argon to prevent material oxidation. The oxygen level in the tent was maintained below 500 ppm as verified by an oxygen analyser. An AMV 4000 arc monitor was used to capture arc current and voltage. The arc current and voltage signals captured by the arc monitor were extracted directly without additional filtering, as the plasma arc process produced stable waveforms without high-frequency noise. Key parameters such as mean current and mean voltage were obtained from the recorded data for each deposition case. A CMOS process camera was used to record the arc behaviour, keyhole characteristics, and metal transfer modes at 55 frames per second, and the videos were manually analysed frame by frame. For each set of process parameters, the deposition reached a steady state to ensure the reliability of the observations.

### Methods

**Fig. 2:**
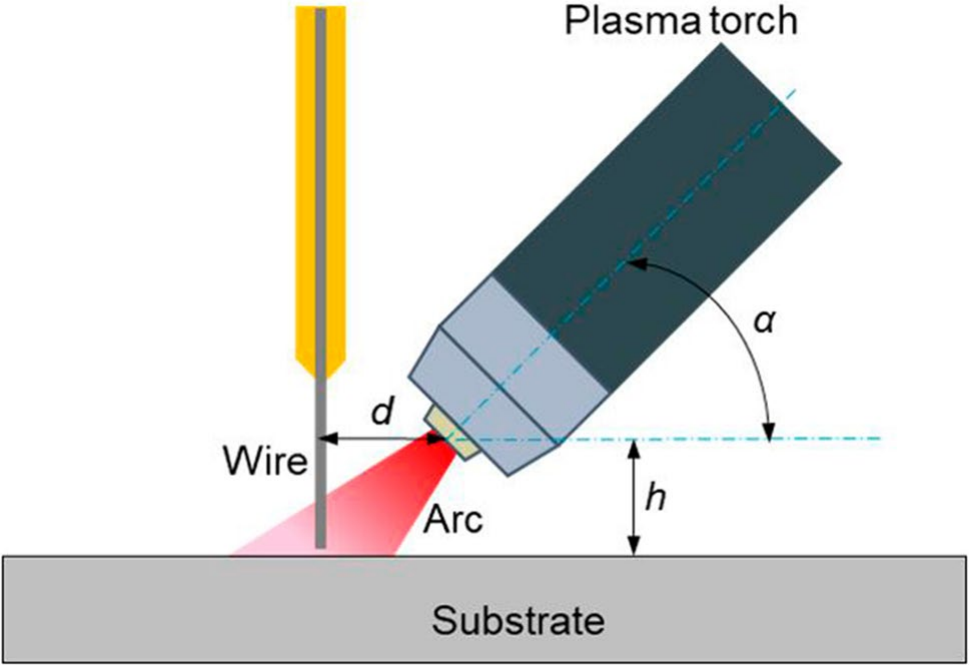
Schematic showing the definition of different parameters for the deposition process

As shown in Fig. 2, the parameters crucial to the process were precisely defined. The distance between the wire and the copper nozzle was defined as *d*, the distance between the copper nozzle and the substrate was defined as *h*, and the torch angle (the angle between the plasma torch and the substrate) was defined as *α*. Based on the trials of the preliminary study, some process parameters were fixed in all the deposition processes in order to obtain acceptable bead quality, as outlined in Table 1.

**Table 1:** Fixed parameters used in all experiments

Parameters	Value
Torch travel speed	4.5 mm/s
Shielding gas flow rate	8 l/min
Bead length	100 mm
Copper nozzle to substrate stand-off (*h*)	8 mm
Copper nozzle diameter	3.9 mm

In order to study the influence of torch position on bead shape, three different positions were systematically examined, including torch leading, torch trailing, and torch positioned at the side. The process parameters used in these three cases, including the torch angle (*α*), arc current, WFS, wire-to-nozzle distance (*d*), and plasma gas flow rate, are shown in Table 2. Following a thorough analysis of the results, as expounded in Sect. 3.1, the travel direction characterised by the torch trailing was selected as the optimal configuration for all subsequent experiments.

**Table 2:** Parameters used for the analysis of torch position effect

Parameters	Value
Torch angle (*α*)	45°
Arc current	200 A
WFS	2.5 m/min
Wire-to-nozzle distance (*d*)	7.5 mm
Plasma gas flow rate	0.8 l/min

More deposits were conducted with both the new and the conventional configurations to compare the keyhole behaviour and the maximum wire-feeding rate that can be achieved. For the analysis of keyhole behaviour, a constant arc current of 200 A and a WFS of 1.5 m/min were used for both configurations. For the analysis of the maximum feeding rate that can be achieved, the WFS was increased from a low value to an upper limit value where no more wire can be melted as identified by the process camera.

To evaluate the metal transfer behaviour obtained with the new configuration, a systematic exploration of various process parameters was conducted. The comprehensive set of parameters investigated is outlined in Table 3. Amongst these experimental cases, the effect of WFS on metal transfer was studied by comparing the results from Cases 1 to 3. Similarly, the effects of arc current (Cases 3–5) and plasma gas flow rate (Cases 3, 6, 7) on metal transfer were also studied.

**Table 3:** Parameters used for the analysis of metal transfer behaviour

Case	WFS (m/min)	Current (A)	Plasma gas flow rate (l/min)	Torch angle (°)	Wire-to-nozzle distance (mm)
1	3.5	200	0.8	30	5
2	3	200	0.8	30	5
3	2.5	200	0.8	30	5
4	2.5	180	0.8	30	5
5	2.5	160	0.8	30	5
6	2.5	200	0.7	30	5
7	2.5	200	0.6	30	5

### Numerical simulation

To gain insight into the bead formation mechanism in the new configuration, a multi-physics simulation was also performed based on our recently reported wirefeeding model [22]. The heat transfer and fluid flow behaviour using the torch leading and torch trailing positions were simulated. In the simulations, the vertical wire feeding was described through the mixture theory and Euler method [22, 23]. The liquid–solid interface was tracked implicitly using the enthalpy–porosity technique [24]. The free surface was tracked utilising the volume of fluid (VOF) method [25]. A surface heat source with an ellipsoid shape was assumed to simulate the inclined arc. The centre of the heat source was located at the extension line of the inclined tungsten centre. The shading effects caused by the wire on arc energy and pressure were also considered in the model. Detailed information on the wire-feeding model, including boundary conditions and numerical methods can be found in Ref. [22].

In this study, the experimental tests and numerical simulations served distinct but complementary purposes. The experimental tests were designed to directly observe and characterise the keyhole behaviour, bead morphology, and metal transfer dynamics under different process conditions. These results provided empirical evidence for evaluating the effectiveness of the new configuration, optimising process parameters, and identifying the practical limits. In contrast, the numerical simulations aimed to reveal the underlying physical mechanisms governing bead formation and fluid flow phenomena, which are difficult to measure experimentally. The objective of this study is not to systematically optimise process parameters, but rather to investigate the fundamental mechanisms governing metal transfer, bead formation, and keyhole behaviour under the new configuration. The findings are intended to deepen the understanding of this novel process and to lay a foundation for future optimisation studies.

## Results and discussion

### Effect of torch position on bead formation

**Fig. 3:**
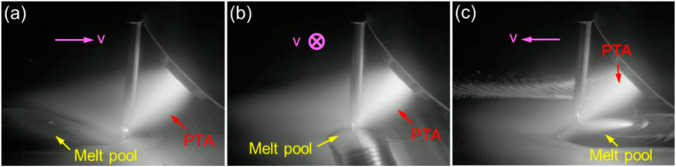
Deposition processes with different torch positions (travel direction indicated by the pink arrows): **a** torch leading, **b** torch at the side, and **c** torch trailing

**Fig. 4:**
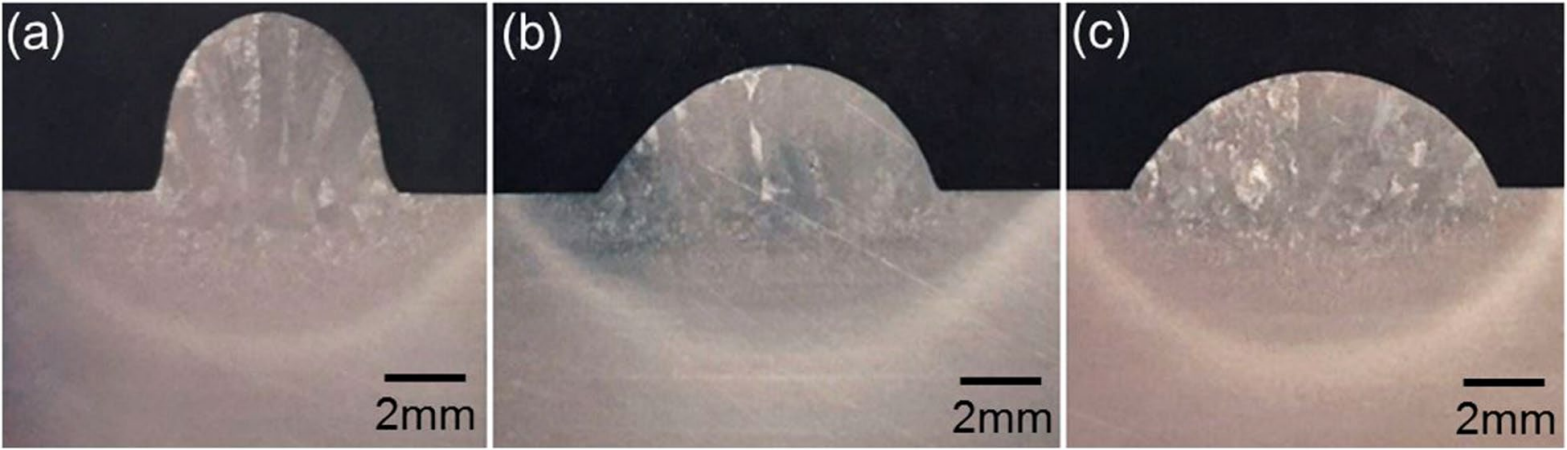
Cross-sections of the beads obtained with different torch positions: **a** torch leading, **b** torch on the side, and **c** torch trailing

The processes with the proposed ‘inclined torch plus vertical wire’ and the conventional ‘vertical torch plus inclined wire’ will be referred to as the new configuration and conventional configuration, respectively. Figure 3 shows the deposition process with the new configuration in different torch positions, and Fig. 4 shows the corresponding cross-sections of the deposited beads. The wire could be fully melted in all three cases (Fig. 3); the obtained bead shapes, however, are different, as seen in Fig. 4. The bead achieved in the torch leading case is narrower and taller than those in the other two cases, with the exact values provided in Table 4. It can be clearly seen from Fig. 3a that in the torch leading case, the PTA impacts on the melt pool, where the arc pressure pushes the melt pool backwards, making it taller. However, in the other two cases (Fig. 3b, c), the PTA is in very little contact with the melt pool, thereby the arc pressure having relatively low effect on the fluid flow. In addition, Fig. 4b shows that the bead obtained with the torch at the side exhibits an asymmetric shape. This is primarily caused by the arc pressure blowing the molten material to the left side.

**Table 4:** Bead geometry obtained with different torch positions

Torch position	Bead width (mm)	Difference compared to Case 3	Bead height (mm)	Difference compared to Case 3
Case 1—torch leading	6.0	− 34.07%	4.5	55.17%
Case 2—torch on the side	8.4	− 7.69%	3.2	10.34%
Case 3—torch trailing	9.1	NA	2.9	NA

**Fig. 5:**
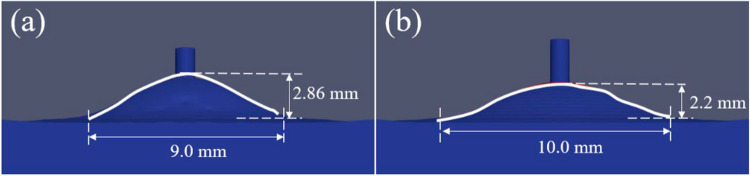
Cross-section of the simulated beads obtained with two different torch positions: **a** torch leading and **b** torch trailing

**Fig. 6:**
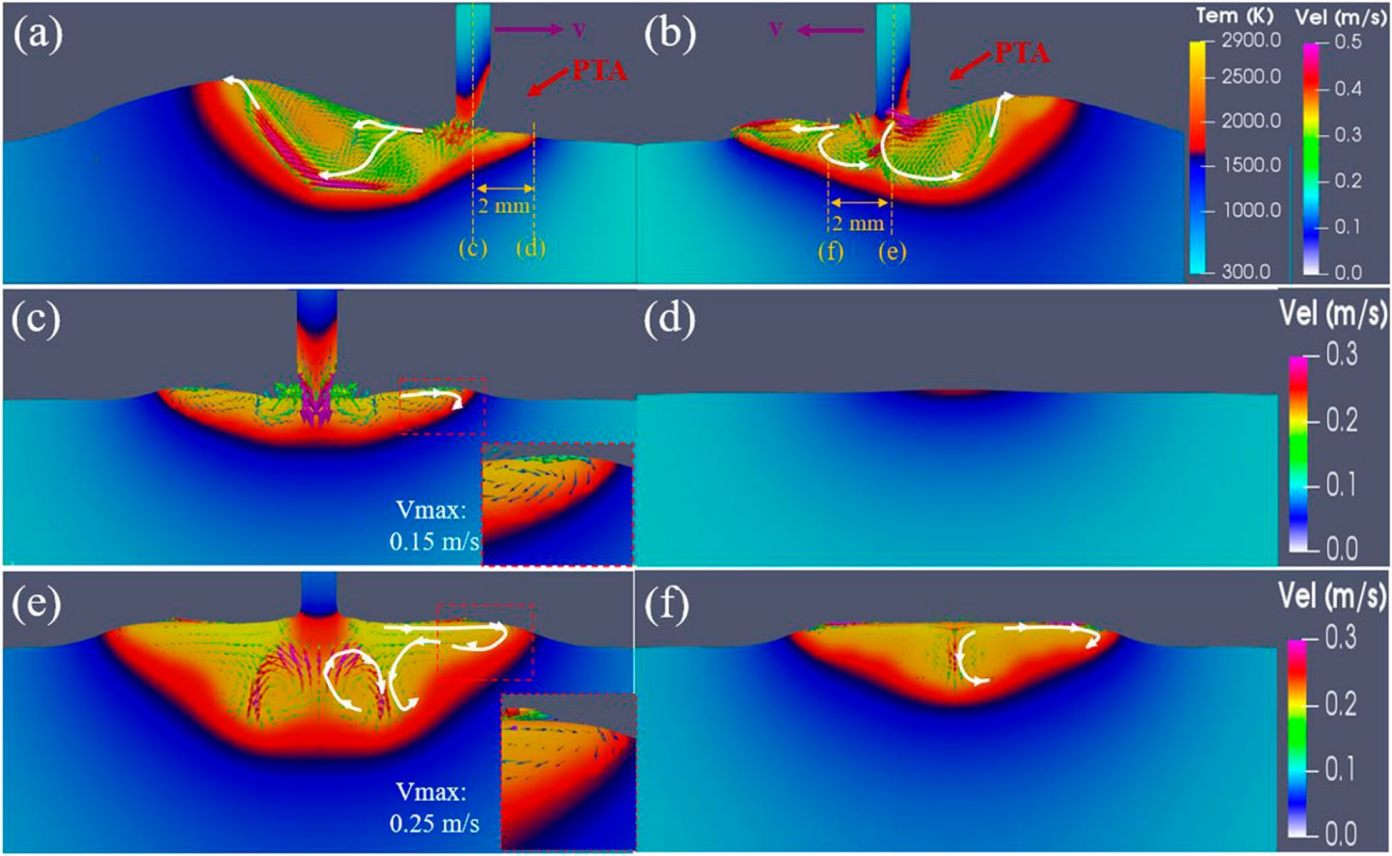
Temperature and velocity distributions in the melt pool incorporated with the metal transfer process: the central longitudinal slices in the torch leading (**a**) and trailing (**b**) cases; the lateral slices right at **c** and 2 mm before (**d**) the wire centre in the torch leading case, and right at **e** and 2 mm before (**f**) the wire centre in the torch trailing case

Figure 5 shows the simulated bead geometries obtained with the torch leading and torch trailing positions. Consistent with the experimental results, the simulated results show that a taller and narrower bead is achieved with the torch leading position. The heat transfer and fluid flows in the melt pool were analysed to understand the bead formation in the two cases, as shown in Fig. 6. In the torch leading case (Fig. 6a), the vertical filler wire was melted and blown by the inclined arc. When the melted wire touched the melt pool, a temporary liquid bridge was formed, and strong downward and backward flows with velocity about 0.45 m/s occurred, driven by the surface tension of the melt pool and arc forces. The liquid metal then was transferred from wire to the melt pool. Driven by the backward-inclined arc pressure, arc shear stress and Marangoni shear stress, significant backward and downward flows transported the liquid metal to the rear of the melt pool, resulting in a relatively large layer height. In the torch trailing case, it is easy to know that the vertical filler wire was also melted and blown by the inclined arc. When the wire touched the melt pool, as shown in Fig. 6b, a liquid bridge was also formed. Strong downward and forward flows with a velocity of about 0.5 m/s occurred, driven by surface tension of the melt pool and arc forces. By the effect of the forward and downward arc pressure and shear stress, the liquid metal transferred from the wire to the melt pool was first transported to the front and then to the rear of the melt pool. These forward surface flows reduced the metal accumulation at the rear of the melt pool, leading to a significantly lower layer height compared to that obtained in the torch leading case, which is in general desirable in AM as it helps achieve a smoother surface with reduced waviness.

The difference in bead formation between the two cases can also be explained by examining the lateral velocity and temperature distribution in the melt pool, as shown in Fig. 6c–f. In the torch leading case, the lateral flows at the edge of the melt pool are relatively mild at the wire centre, with a maximum velocity of about 0.15 m/s (Fig. 6c). The substrate 2 mm before the wire centre (also the arc centre) was not melted yet (Fig. 6d). In comparison, in the torch trailing case, a significant melt pool was already formed at 2 mm ahead of the wire centre (Fig. 6f) because of the convective heat carried from the liquid metal flows driven by the forward arc forces. Thus, more heat was transferred laterally when the forward flows moved back to the rear melt pool, resulting in a wider bead width compared to that in the torch leading case. As shown in Fig. 6e, considerable lateral flows with a maximum velocity of about 0.25 m/s occurred at the wire centre slice, which is significantly larger than that in the torch leading case. This also demonstrates that a wider bead can be obtained in the torch trailing case.

In WAAM, the geometry of a single bead is an important factor, which determines the surface waviness of a deposited component. A flatter bead results in a lower surface waviness, meaning less material needs to be machined off to meet the final surface requirement [11]. In addition, a symmetric bead shape is required when building real components. Therefore, the bead obtained with the torch trailing position is desired amongst all the three cases studied.

### Keyhole behaviour and deposition rate

#### Keyhole behaviour

**Fig. 7:**
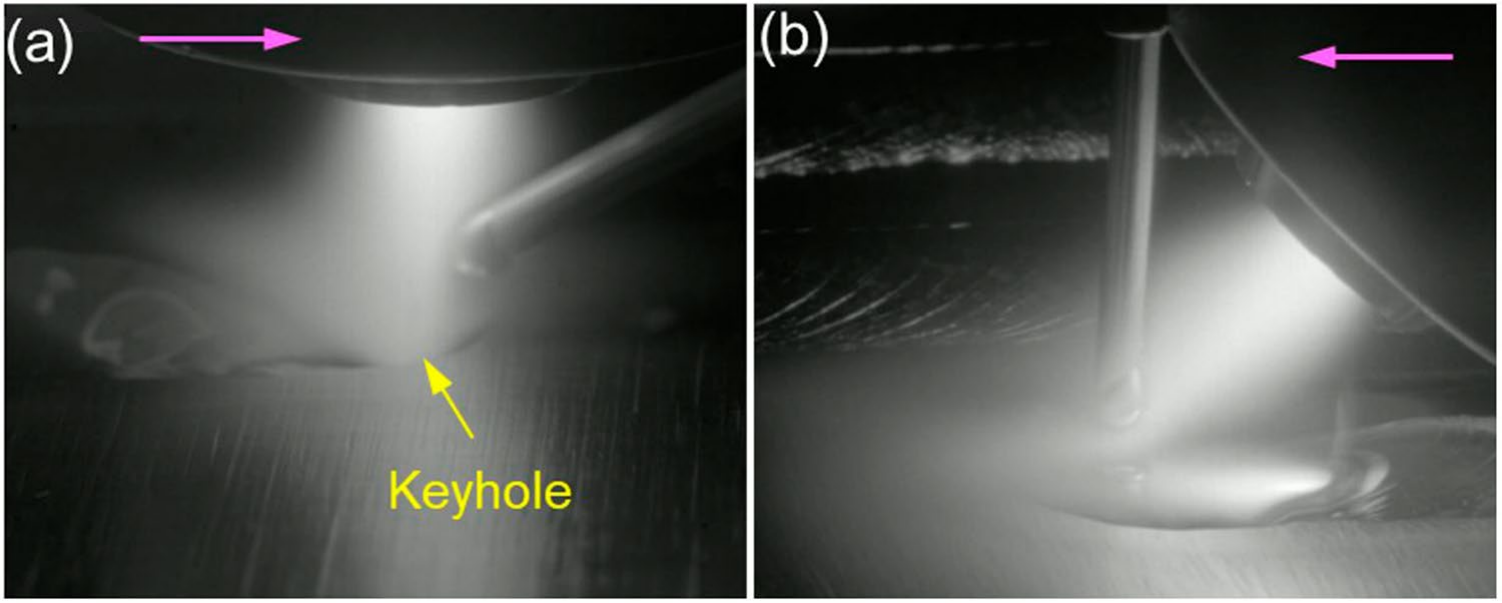
A comparison of the PTA-based deposition process between using the **a** conventional configuration and **b** new configuration

Figure 7 shows a comparison of the deposition process between using the conventional and new configurations with the same process parameters used. One can see that there is a large depression in the melt pool using the conventional configuration with a vertical torch and an inclined wire (Fig. 7a), leading to a high risk of creating a keyhole defect in this scenario, whereas there is no depression in the melt pool using the new configuration with a vertical wire and an inclined torch (Fig. 7b), showing a very low potential of creating a keyhole defect in this case.

It has been reported that in PTA-based processes, keyhole formation is caused by a combination of energy input and arc pressure, where the energy input melts the material and the arc pressure creates a large depression in the melt pool [15, 26–28]. The energy inputs in the two studied cases were the same, and therefore, the keyhole generated in the conventional configuration case was caused by a higher arc pressure compared to that in the new configuration case. In fact, the arc pressure acting directly on the workpiece is expressed as follows:

1 $$\rho_n=\rho\sin\alpha$$

where ${p}_{n}$ is the arc pressure normal to the workpiece, whilst $p$ is the total arc pressure and $\alpha$ is the torch angle. Therefore, the arc pressure acting directly on the workpiece increases as the torch angle increases from 0 to 90°. For example, at a torch angle of 30°, the arc pressure acting directly on the workpiece will be reduced by 50% compared to that using a vertical torch. In addition, at the same torch stand-off distance, the arc column is longer with the inclined torch compared to that with a vertical torch, which will also reduce the arc pressure acting on the workpiece. Therefore, with the same process parameters, the new configuration results in a lower arc pressure exerted on the workpiece compared to that with the conventional configuration, leading to a lower likelihood of keyhole formation in the former case.

**Fig. 8:**
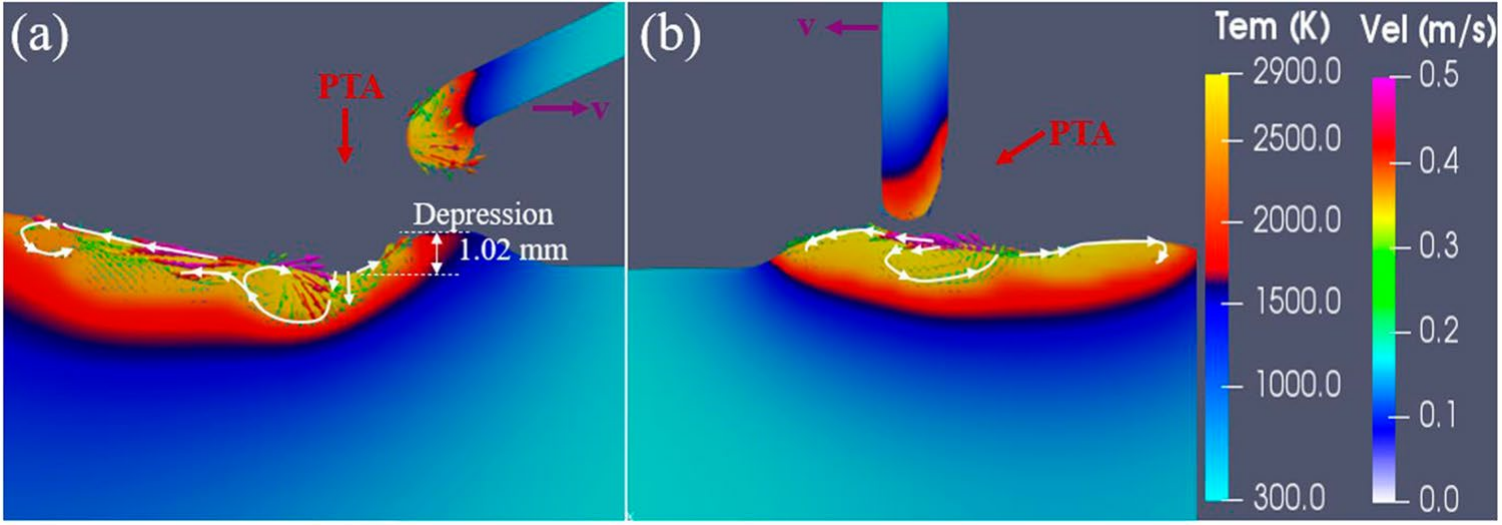
A comparison of simulated melt pool in the PTA-based deposition process between using the **a** conventional configuration and **b** new configuration

Figure 8 shows the longitudinal central section of the simulated melt pool using the conventional configuration and new configuration. In the conventional configuration, downward flows occurred at the arc centre driven by the strong arc pressure generated from the vertical torch (Fig. 8a). Thus, a significant depression (1.02 mm) was formed there. The liquid metal was transported to the rear melt pool by the backward flows primarily driven by Marangoni shear stress, droplet impaction, and arc shear stress [22]. However, in the new configuration, strong forward flows with a slightly downward direction occurred with the effect of inclined arc pressure and arc shear stress, Marangoni shear stress, and droplet impaction (Fig. 8b). No significant depression occurred, and the flows in the melt pool were different from those in the conventional configuration. The simulations further demonstrate that the new configuration changes the flow behaviours in the melt pool and suppresses keyhole formation.

#### Deposition rate

**Fig. 9:**
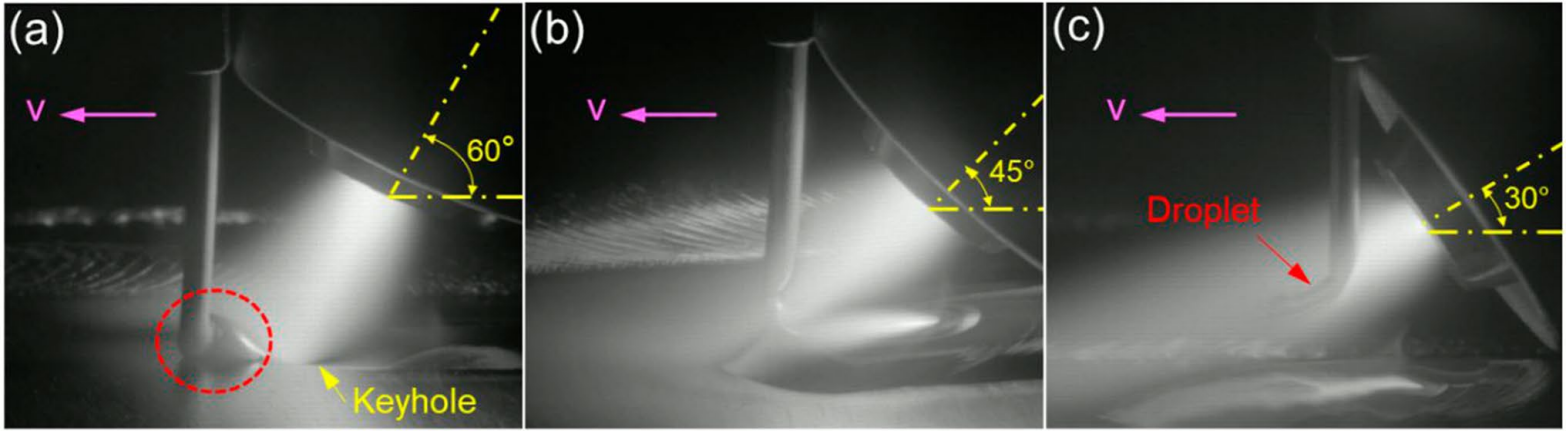
Deposition processes at different torch angles: **a** 60°, **b** 45°, and **c** 30°

Figure 9 shows the deposition processes with different torch angles. Due to the lack of access, in each case, the wire was positioned as close as practically possible to the plasma torch, which resulted in different distances (*d*) between the wire and copper nozzle. The distances (*d*) at torch angles of 30°, 45°, and 60° were 5, 7.5, and 10 mm, respectively. This led to different wire melting behaviour. At a torch angle of 60° (Fig. 9a), the wire could not reach the PTA centre, leading to a partially melted wire and an unstable process. In addition, a keyhole was formed in this case. When the torch angle was reduced to 45° (Fig. 9b), the wire could not be fully melted by the PTA before reaching the melt pool. This means that the wire took additional energy from the melt pool to be fully melted. At a torch angle of 30° (Fig. 9c), the wire was fully melted by the PTA before reaching the melt pool, and the metal transfer turned from a surface tension mode (Fig. 9b) into a free space droplet mode.

The difference in melting behaviour in the above discussed cases was mainly attributed to the different positions of the wire that reached in the arc column. It is known that there is a steep temperature gradient in the PTA column with the temperature reaching the highest point near the cathode [16]. Therefore, the closer the wire is to the copper nozzle (or cathode), the easier the wire can be melted. In the three cases shown in Fig. 9, the wire could be melted entirely by the PTA in the last case due to the wire being in the closest proximity to the arc column, hence reaching the hottest region and achieving the highest melting efficiency. Therefore, a torch angle of 30° was used in the following experiments.

**Fig. 10:**
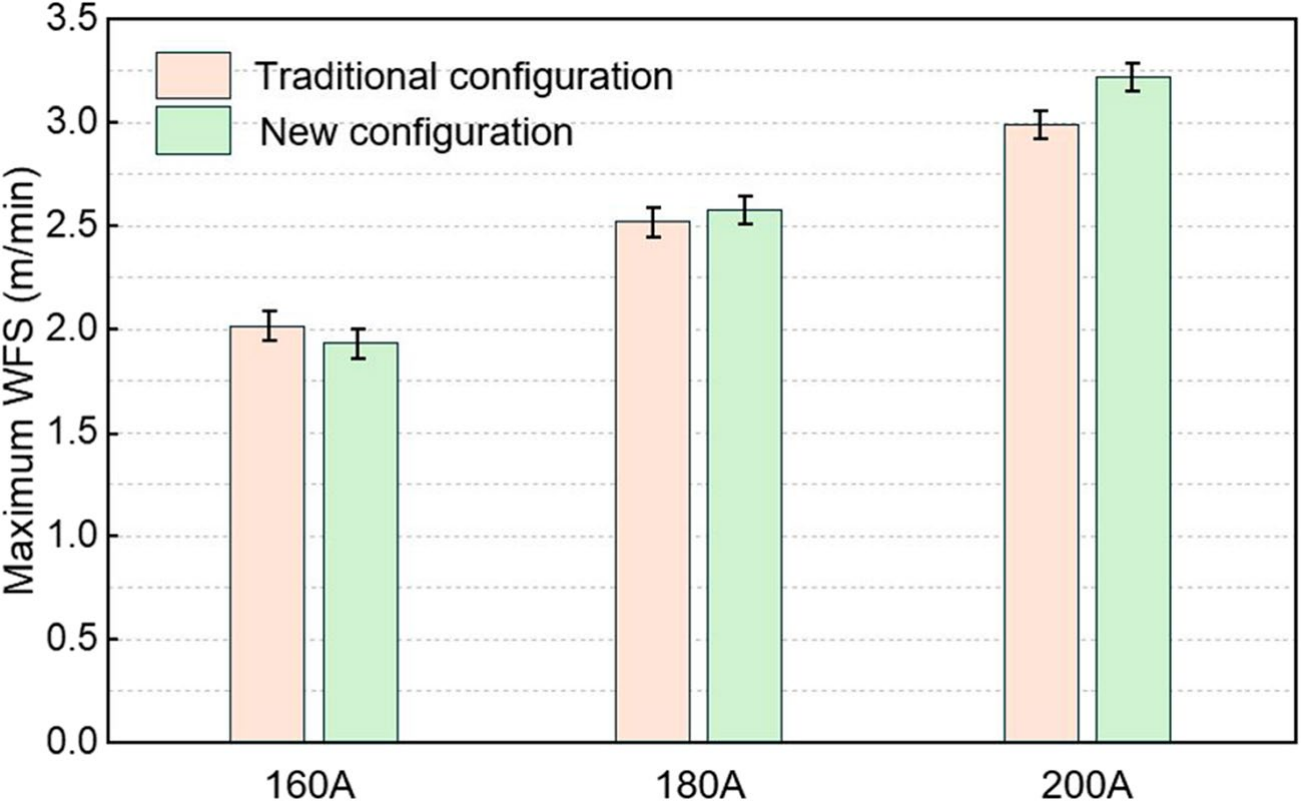
Maximum wire-feeding rates obtained with the new and conventional configurations at different levels of arc current

Figure 10 presents the maximum wire-feeding rates achieved with the conventional and new configurations at different arc current levels. At currents of 160, 180, and 200 A, the maximum wire-feeding rates for both configurations were similar, reaching approximately 2.0, 2.5, and 3.0 m/min, respectively. Furthermore, the maximum wire-feeding rate increased proportionally with the energy input, which is consistent with the findings reported in Ref. [11]. Regardless of the configuration used in this study, similar deposition rates will be achieved as long as the energy input and the relative position of the wire in the arc column are the same.

### Metal transfer and bead formation

#### Metal transfer under different processing conditions

**Fig. 11:**
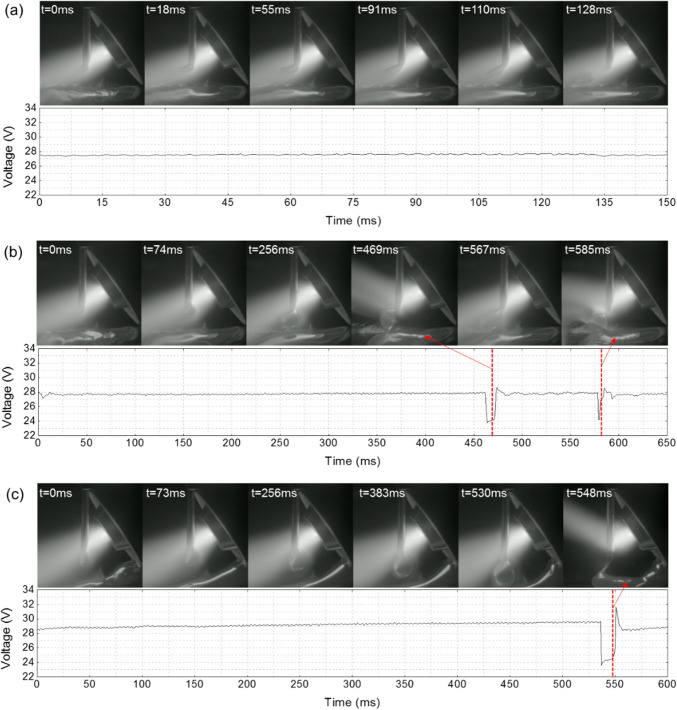
Droplet transfer and voltage waveform at different WFSs: **a** 2.5 m/min, **b** 3.0 m/min, and **c** 3.5 m/min

Figure 11 shows the metal transfer and the corresponding voltage waveform at different WFSs. At a relatively low WFS of 2.5 m/min (Fig. 11a), the wire was completedly melted by the PTA, and the droplets were small when detaching from the wire tip. The droplet transfer period was 128 ms in this case, and the voltage was stable throughout the entire transfer phase. When the WFS was increased to 3.0 m/min (Fig. 11b), the droplets became larger. The droplet transfer period increased to 585 ms, marking a fourfold increase compared to the first case. It is noteworthy that between the time interval of 469 and 585 ms, the droplet contacted the melt pool, inducing a noticeable drop in the arc voltage from 28 to 24 V due to the shortening of the arc column. This means that the droplets were large enough to bridge the arc gap, providing an easier path for current flow from the electrode to the workpiece. When the WFS was increased to 3.5 m/min (Fig. 11c), the wire could not be completely melted by the PTA and passed across the entire arc column to the other side. This resulted in a slow accumulation of the liquid metal and the growth of large droplets at the wire tip.

At a low WFS, the droplets detached from the wire in the middle of the arc column where the arc pressure acted as a dominant detachment force that could overcome the surface tension. However, at a high WFS, the temperature of the droplets will be lower, and the surface tension of the droplets will be higher, leading to the wire being more difficult to melt. The wire could only be fully melted after passing the hottest part of the arc column. In this scenario, the arc pressure around the wire tip was notably lower compared to that in the arc centre. This reduced arc pressure was insufficient to detach droplets from the wire, allowing them to grow to substantial sizes until making contact with the melt pool. The wire melting and metal transfer behaviour play a critical role in the bead formation, which will be discussed later in Sect. 3.3.4.

**Fig. 12:**
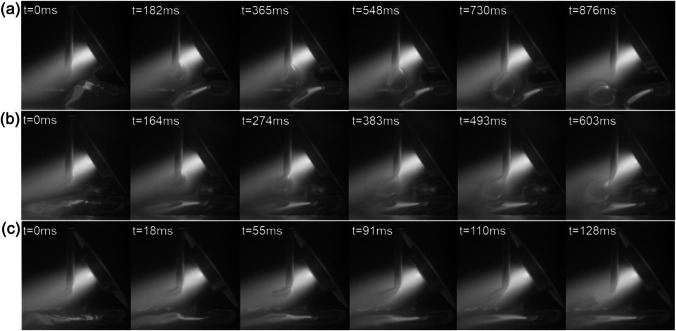
Droplet transfer at different levels of current: **a** 160 A, **b** 180 A, and **c** 200 A

Figure 12 shows the effect of arc current on the metal transfer. At a relatively low current of 160 A (Fig. 12a), large droplets were formed along with a relatively long transfer period of 876 ms. This is because the surface tension of the droplets was high due to the relatively low temperature (or energy input) and the arc pressure was low at a relatively low arc current. Consequently, the arc pressure could not detach the droplets from the wire tip very easily, and the wire passed across the entire arc column. At an arc current of 180 A (Fig. 12b), the wire could be melted more easily due to the increased energy input. The increased arc pressure and decreased surface tension facilitated easier detachment of droplets from the wire tip, as reflected by the smaller droplet size and shortened transfer period (603 ms). When the arc current was increased to 200 A, the high arc pressure and low surface tension led to a significant decrease in both droplet size and transfer period (128 ms), as shown in Fig. 12c.

**Fig. 13:**
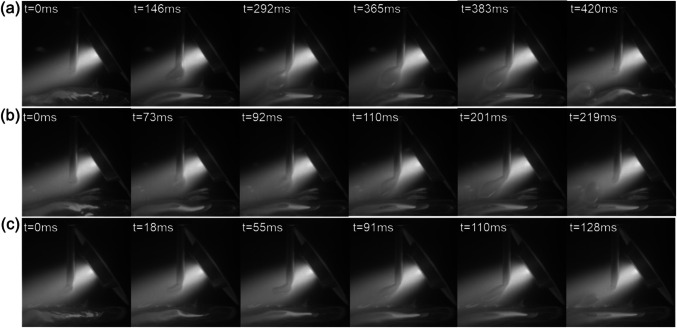
Droplet transfer with different plasma gas flow rates: **a** 0.6 l/min, **b** 0.7 l/min, and **c** 0.8 l/min

Figure 13 shows the effect of plasma gas flow rate on the metal transfer behaviour. At a plasma gas flow rate of 0.6 l/min (Fig. 13a), the droplets were relatively large with a transfer period of 420 ms. When the plasma gas flow rate was increased to 0.7 l/min (Fig. 13b), the droplets became smaller, and the transfer period reduced to 219 ms. When the plasma gas flow rate was further increased to 0.8 l/min (Fig. 13c), much smaller droplets and a higher frequency were observed. The arc pressure increases with the increasing plasma gas flow rate due to the increased plasma drag force [11]. At a low plasma gas flow rate, the relatively low arc pressure struggled to detach droplets, allowing them to attain significant sizes. The droplets could only be detached when they were in contact with the melt pool, leading to large droplets and a low transfer frequency. However, at a high plasma gas flow rate, the high arc pressure had enough force to dislodge the droplets from the wire tip before they could grow up to a significant size, resulting in a high droplet transfer frequency.

**Fig. 14:**
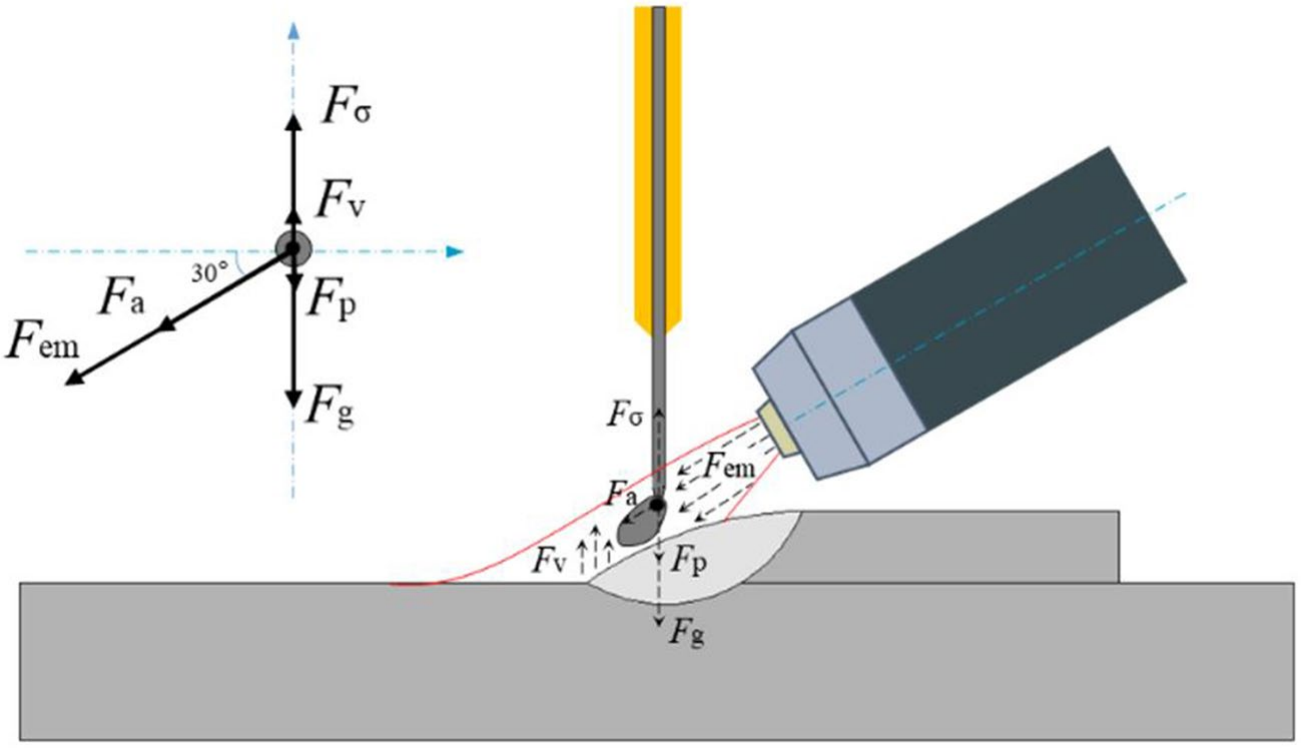
Forces acting on a droplet in the PTA deposition process with a vertical wire and inclined torch (*α* = 30°)

There are different forces acting on a droplet in metal transfer, and the detachment of a droplet is driven by the balance of all the forces acting on it. Figure 14 schematically shows the forces acting on a droplet in the PTA deposition process with the new configuration, where ${F}_{g}$ is the gravitational force, ${F}_{em}$ is the electromagnetic force, ${F}_{a}$ is the plasma drag force,${F}_{p}$ is the driving force of the wire, ${F}_{v}$ is the vapour jet force, and ${F}_{\sigma }$ is the surface tension. Due to the relatively low WFS used and low volume of metal vapour produced in WAAM, the driving force of the wire and the vapour jet force can be neglected. In addition, the arc pressure is mainly composed of electromagnetic force and plasma drag force. Therefore, the main forces acting on the droplets are arc pressure, surface tension, and gravitational force. The metal transfer behaviour, including the droplet size, droplet transfer frequency, and droplet movement direction are all dependent on the resultant force acting on the droplet as demonstrated earlier.

#### Bead formation

**Fig. 15:**
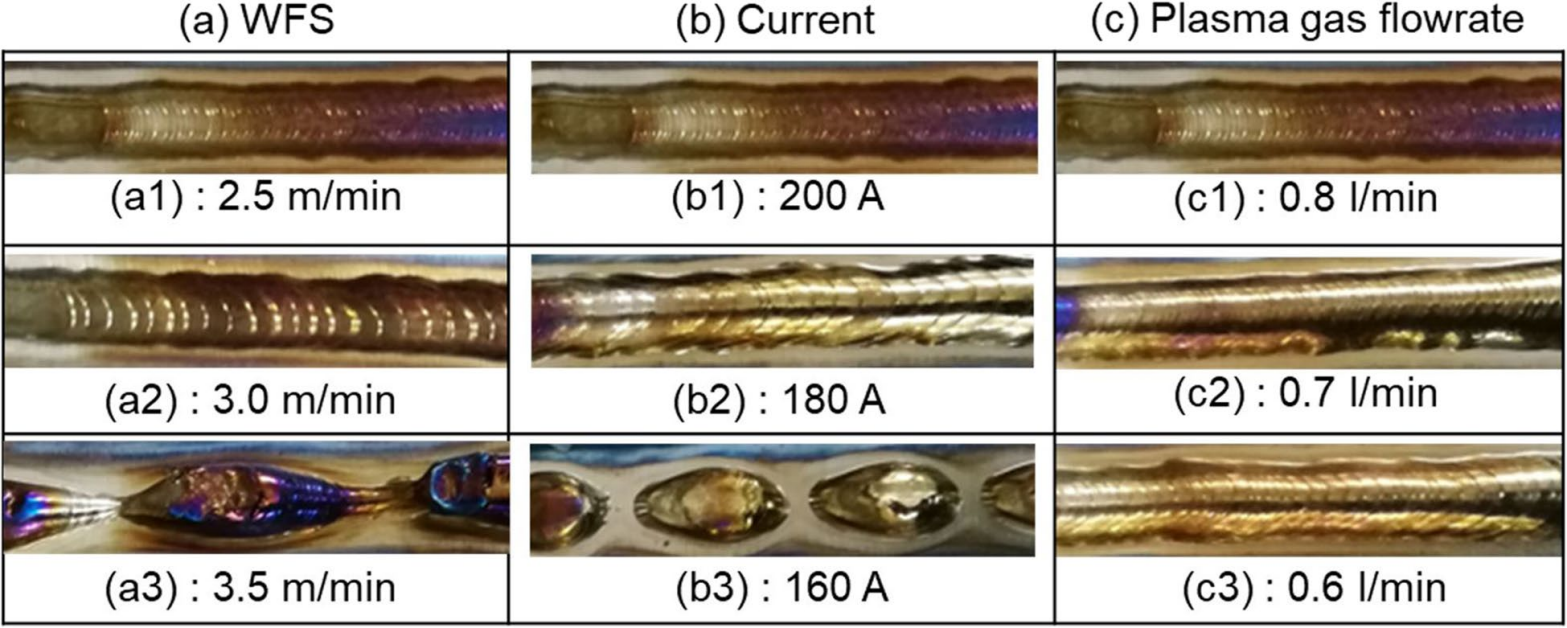
Bead appearance as a function of different process parameters: **a** WFS, **b** current, and **c** plasma gas flow rate. All parameters used in each case are seen in Table 3

Figure 15 shows the bead appearance obtained with the corresponding processes as presented in Figs. 11, 12, and 13. In Fig. 15a, periodic ripples are observed on the bead surface, and they become larger as the WFS increases from 2.5 to 3.0 m/min due to the decreased droplet transfer frequency. At a WFS of 3.5 m/min, the energy input was insufficient to generate a continuous melt pool, resulting in disconnected beads on the substrate. Figure 15b shows that reducing arc current has a similar effect on the bead appearance as increasing WFS. As the current decreases from 200 to 180 A, the droplets become larger, and the transfer frequency becomes lower, resulting in larger ripples on the bead surface. At a current of 160 A, disconnected beads occurred due to the low energy input. In Fig. 15c, the decreased plasma gas flow rate has a little effect on the bead appearance due to the minor variation in droplet transfer behaviour, as can be seen in Fig. 13.

**Fig. 16:**
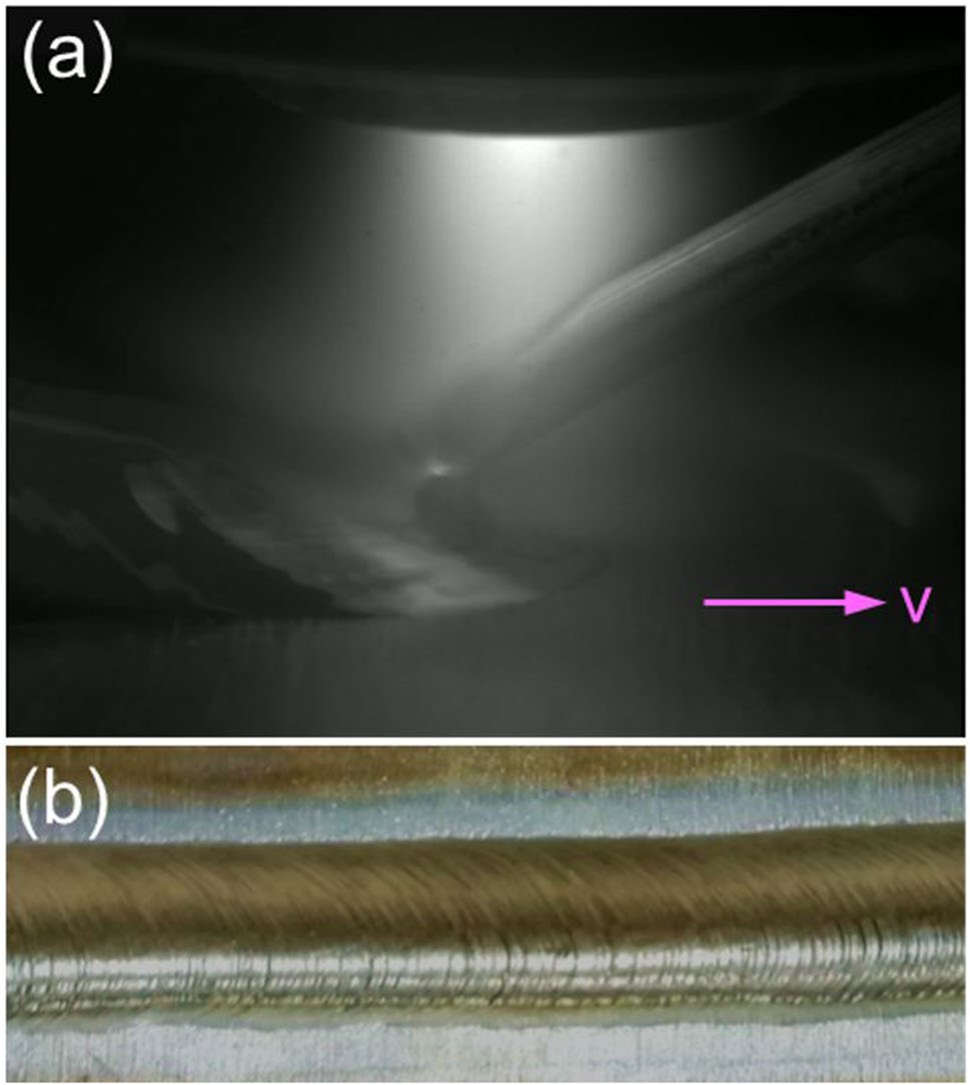
The **a** deposition process and **b** bead appearance obtained with the conventional configuration at a current of 200 A, a WFS of 2.5 m/min, and a plasma gas flow rate of 0.8 l/min

One can see that the beads obtained with the new configuration exhibit relatively low surface quality. Figure 16 shows a typical deposition process with the conventional configuration and the resulting bead appearance. All the parameters used in this case were the same as those in Fig. 15(a1). The bead obtained with the conventional configuration is more uniform compared to that obtained with the new configuration. In PTA-based WAAM, the bead quality is largely dependent on the metal transfer behaviour. With the new configuration, the movement direction of the droplets is determined by the net effect of arc pressure and gravitational force, which is not constant and can vary with many process parameters. By contrast, with the conventional configuration, the directions of both arc pressure and gravitational force are normal to the workpiece, making the droplets move to the melt pool vertically. Therefore, compared to that with the new configuration, the droplets obtained with the conventional configuration move to the melt pool without the risk of being blown away. Consequently, the beads obtained with the conventional configuration have a more uniform and regular geometry than those obtained with the new configuration.

Low bead uniformity increases surface waviness in printed components, meaning more material must be machined away to meet the final surface quality requirements. Even worse, in some cases, poor bead uniformity can lead to defects such as lack of fusion, rendering the part unacceptable. To improve bead quality, it is crucial to control the metal transfer behaviour, including the size, frequency, direction, and mode of transfer, thereby achieving a uniform bead appearance and high overall quality. Furthermore, to enhance the omnidirectional deposition capability of the process, it is essential to optimise key parameters such as the plasma gas flow rate, wire-feeding angle, and torch inclination. Adjusting the plasma gas flow rate directly influences arc constriction and stiffness, which in turn affects the arc pressure distribution and fluid flow behaviour within the molten pool. In addition, careful coordination of the wire-feeding angle and torch inclination can significantly improve the consistency of bead formation in different deposition directions. Further investigations are needed to fully explore and validate these optimisation approaches.

Based on the above results and discussion, it can be concluded that the new configuration presents a combination of advantages and disadvantages when compared to the conventional configuration. The implementation of an inclined plasma torch in the new configuration leads to a direct reduction in the arc pressure exerted on the workpiece, thereby diminishing the likelihood of keyhole formation. However, in the new configuration, the movement direction of the droplet is determined by the resultant force acting on it, meaning that the droplet transfer is sensitive to many process parameters. In general, the beads obtained with the new configuration have lower uniformity than those obtained with the conventional configuration. Moreover, the deposition process with the new configuration is not omnidirectional due to the influence of arc pressure, and different bead shapes were achieved with different torch positions. The best bead quality characterised by good flatness and symmetric shape was obtained with the torch trailing position. This is similar to the conventional configuration where a torch trailing position is also commonly used because it gives high process tolerance and good bead quality in the deposition process.

## Conclusions

In this study, a PTA-based WAAM process employing a vertical wire and an inclined torch was investigated and systematically compared with the conventional configuration using an inclined wire and vertical torch. The key findings are summarised as follows:

1. The new configuration significantly reduces the likelihood of keyhole formation due to a lower arc pressure directly acting on the workpiece. This improvement enhances the process stability and offers the potential for employing higher arc currents and wire feed speeds to achieve increased deposition rates.
2. Metal transfer behaviour in the new configuration is strongly influenced by the interplay of arc pressure, gravitational forces, and surface tension, which are modulated by process parameters. An increase in WFS or a reduction in arc current and plasma gas flow rate leads to the formation of larger droplets and a decrease in droplet transfer frequency.
3. In contrast to the conventional configuration, where droplets are consistently directed vertically into the melt pool by arc pressure, the new configuration results in more variable droplet trajectories due to the competing effects of arc pressure and gravity. Consequently, bead uniformity is slightly reduced compared to the conventional process.
4. The wire-feeding position relative to the arc column plays a critical role in wire melting efficiency, attributed to the asymmetric energy distribution of the PTA. Wire melting is more effective when the wire is positioned closer to the cathode. Under identical arc current and wire-feeding conditions, the maximum achievable deposition rates for the new and conventional configurations are comparable.
5. Due to the altered arc pressure distribution, bead formation in the new configuration exhibits sensitivity to the torch position, resulting in non-omnidirectional deposition characteristics. Amongst the tested positions, the torch trailing position yields the most favourable bead geometry, characterised by greater uniformity and symmetry, which is advantageous for part fabrication.

Overall, this study demonstrates the potential and challenges of using a vertical wire and inclined torch configuration in PTA-based WAAM. The findings contribute to a deeper understanding of metal transfer mechanisms and bead formation dynamics under this new configuration. Future work will focus on further optimising torch parameters and wire-feeding strategies to enhance omnidirectional deposition capability and overall build quality.

## Data Availability

Data will be made available on request.
